# Gas sensing response of ion beam irradiated Ga-doped ZnO thin films

**DOI:** 10.1038/s41598-022-26948-8

**Published:** 2022-12-26

**Authors:** R. C. Ramola, Sandhya Negi, Ravi Chand Singh, Fouran Singh

**Affiliations:** 1 Department of Physics, HNB Garhwal University, Badshahi Thaul Campus, Tehri Garhwal, 249 199 India; 2 Department of Physics, Guru Nanak Dev University, Amritsar, 142 005 India; 3 Material Science Group, Inter University Accelerator Centre, New Delhi, 110 067 India

**Keywords:** Materials science, Characterization and analytical techniques

## Abstract

The ion beam induced modified gallium doped ZnO thin films are studied for their gas sensing applications. The Ag^9+^ and Si^6+^ irradiated gallium doped zinc oxide thin films were exposed to various concentrations of ethanol and acetone gas for gas sensing applications. The Ag^9+^ ion irradiated Ga-doped ZnO thin was optimized at different operating temperature. It was observed that gas sensing response for both ethanol and acetone gas increases with increasing Ag^9+^ ion fluence. This indicates that the swift heavy ions have improved the sensitivity of Ga-doled ZnO thin film by reducing the particle size. The Si^6+^ ion irradiated Ga-doped ZnO thin films were also exposed to ethanol and acetone gas for gas sensing applications. In comparison to Ag^9+^ ion irradiated thin film, the film irradiated with Si^6+^ ion beam exhibits a greater sensing response to both ethanol and acetone gas.

## Introduction

The growing interest of research fraternity in the field of gas sensors is because of the necessity of safe human working environment free from poisonous, obnoxious and combustible gases. The volatile organic gases such as acetone, ethanol and ammonia are known as indoor pollutants. The inhalation of these gasses above permissible limit may not be safe for humans. It has therefore become essential to detect the presence of these gases in the human environment^1^. Hence, the major interest is on developing highly sensitive, fast-responding selective gas sensors. Metal oxide semiconductor-based gas sensors are considered one of the best options due to their numerous advantages, including low cost, small size, quick response and recovery time, simple fabrication, and high compatibility with microelectronic processing^2,3^. The discovery of nanotechnology has increased the possibility of reinventing procedures and options for further refinement in terms of sensing response and selectivity of metal oxide-based gas sensors. One of the primary advantages of metal oxide semiconducting nanoparticles is their high surface-to-volume ratio. Because sensing response is strongly dependent on the surface of the materials exposed to gases, the thin film nanostructures-based sensor is expected to outperform the bulk sensor^3^.

Zinc oxide (ZnO) is one of the most prominent metal oxide semiconductors with large exciton binding energy of 60 meV and a wide band energy gap of 3.37 eV at ambient temperature^4^. Because of its abundance in nature and exceptional electrical and optical properties, it has wide a range of applications in electronic and optoelectronic devices such as solar cell window, surface acoustic wave devices and gas sensing applications^5–9^. ZnO thin film is more appropriate for gas sensing applications because of its high surface to volume ratio, allowing adsorption of more gas for surface reactions^10–22^.

Swift heavy ion irradiation (SHI) has created interest among the researchers as a tool for modifying the structural, electrical, and optical properties of materials, thereby greatly improving device efficiency^23–26^. Ion beam irradiation does not change the chemical composition of the material, but it does create structural defects that affect the concentration of adsorption canters and the capacity of adsorption on the material surface. It is therefore possible to tailor the structural, electrical, and optical properties of the target materials by varying the energy and fluence of the incident ion beam^26,27^.

The objective of this study is to explore the procedures for developing a highly sensitive gas sensor for ethanol and acetone vapours. The response and recovery time, sensitivity, and selectivity of Ga doped ZnO thin films modified by ion beam irradiation have been analyzed for this purpose.

## Experimental methods

Sol–gel spin coating method was used to deposit Ga-dopped ZnO thin films-based metal oxide semiconductors on quartz substrates. The deposited films were modified by swift heavy ion irradiation. Ion beam modification of Ga-doped ZnO thin films were performed using 120 MeV Ag^9+^ and 70 MeV Si^6+^ ions. The range of incident ions in the ZnO thin film was calculated using the stopping range of ions in matter (SRIM) programme^28^. Free standing Ga-doped ZnO thin films of one square cm were irradiated under high vacuum conditions with 120 MeV Silver and 70 MeV Silicon ions with ion fluences of 5 × 10^13^ ion/cm^2^ and beam current of 1.5 pnA for silver and 1 pnA for silicon beam. The nature of the changes induced by the ion beam has been analyzed by different characterized techniques. Original and irradiated Ga-doped ZnO films were studied for their structural changes using X-ray diffraction (XRD). The surface morphology of the films was analyzed by a JEOL JSM-840 scanning electron microscope (SEM).

**Figure 1:**
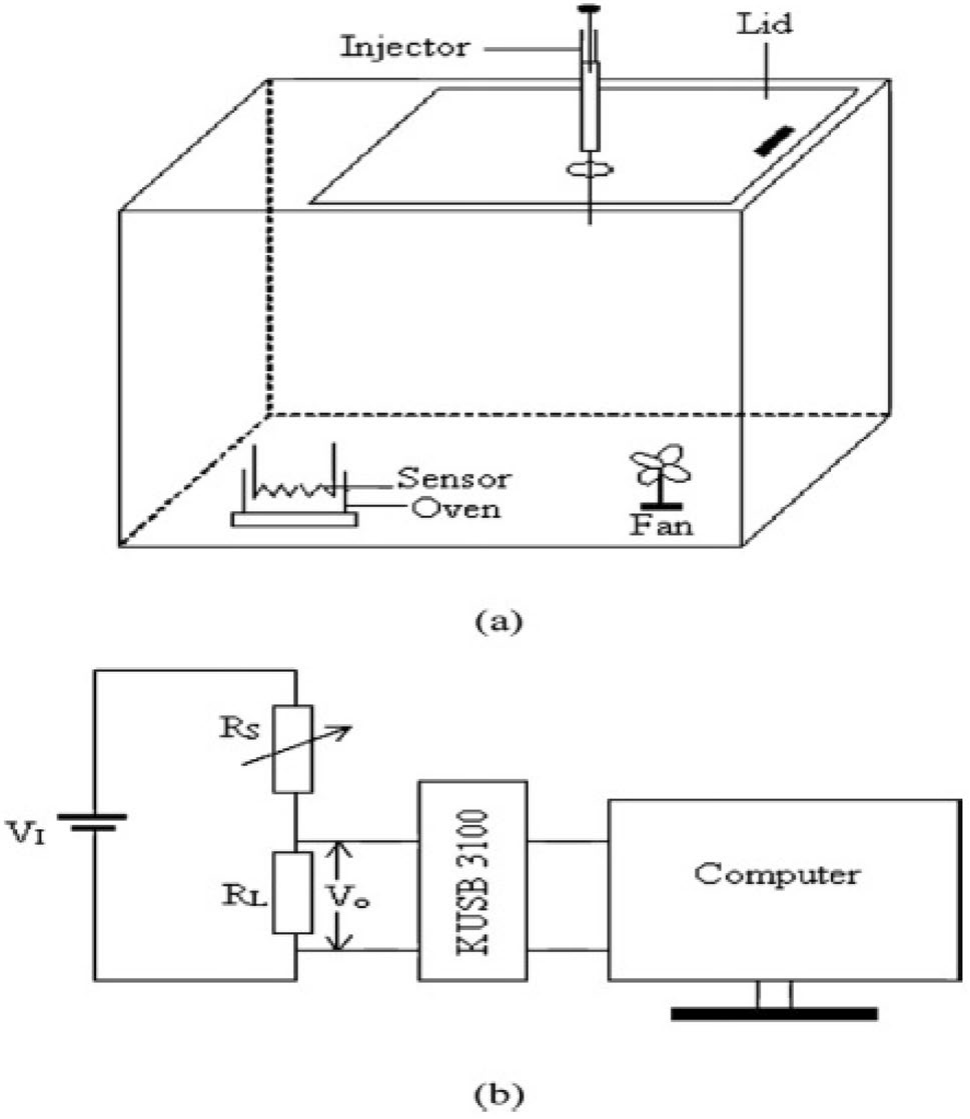
Schematic of sensor unit (**a**) testing chamber (**b**) data acquisition system.

The response of gas sensor was measured using an apparatus^29^ consisting of a simple potentiometer arrangement in a chamber with sample holder, a temperature controlled oven and a circulating fan (Fig. 1).

The sensor was placed in the oven at an appropriate temperature and known quantities of ethanol and acetone gases were injected into the chamber. The real time voltage signals across the load resistance, R_L_, connected in series with the sensor were recorded with Keithley Data acquisition Module KUSB-3100 connected with a computer. The magnitude of sensor response was determined as the ratio of the sensor resistances (R_a_/R_s_) in air-gas mixture and air ambience^30–32^. Same procedure was applied to test all sensors for temperatures ranging from 100 to 500 °C. The inter-granular energy conductance of all samples was estimated at 200, 250, 300, 350, 400, 450 and 500 °C. The current (I) flowing through the circuit (Fig. 1b) was measured using the relation V_o_/R_L_. The sensor resistance R_S_ and conductance 1/R_S_ were obtained using the relation V_1_ = I (R_S_ + R_L_). A potential difference of 12 V is applied to the gold coated electrode of Ga doped ZnO thin film, and the potential drop is obtained across the load resistance (R_L_).

The potential drop corresponding to the load resistance will be V_0_ = IR_L._ Using these parameters, the change in the resistance of Ga-doped ZnO semiconductor can be calculated when exposed to varying concentrations of the target gas ethanol and acetone.

1 $$\begin{aligned} V_{1 } & = I\left( {R_{S} + R_{L} } \right) \\ {\text{Or}}\quad V_{1} & = \frac{{V_{0} }}{{R_{L} }}\left( {R_{S} + R_{L} } \right) \\ {\text{Or}}\quad V_{0 } & = \frac{{V_{1} R_{L} }}{{R_{L} + R_{S} }} \\ {\text{So}},\quad R_{S} & = \left( {\frac{{V_{1} R_{L} }}{{V_{0} }}} \right) - R_{L} \\ \end{aligned}$$

The conductance of Ga doped Metal Oxide Semiconductor will be

2 $${\text{S}} = 1/{\text{R}}_{{\text{S}}} = \frac{1}{{\left( {\frac{{V_{1} R_{L} }}{{V_{0} }}} \right) - R_{L} }}$$

## Gas sensing mechanism

### Sensing mechanism of ethanol with Ga-doped ZnO thin film

The gas sensing mechanisms for metal oxide semiconductor sensors are based on the assumption that adsorption of oxygen on the material surface removes some electrons, lowering the conductivity of the material.

When gas molecules come in contact with a material's surface, they interact with oxygen, resulting in inverse charge transference and thus an increase in material conductivity^33^. The variation in gas-induced resistance caused by changes in the height of potential at grain boundaries is utilized to detect ethanol in the air. The grain boundaries contribute to the resistance for polycrystalline substances. The nature of the chemisorbed species also affects the conductivity of the surface of semiconducting oxide crystals, which depends on the concentration of electrons near the material surface. The modification of the surface of zinc oxide film by electron transfer mechanism will also modify the response characteristic of the sensor. The oxidation of the ethanol on the Ga-doped ZnO film surface liberates free electrons and H_2_O and thus decreases its resistance. The atmospheric oxygen is chemisorbed on Ga-doped ZnO film surface as O^2−^ or O^−^. It removes an electron from conduction band of the Ga-doped ZnO film and develops a depletion region on the film surface. The ethanol reacts with chemisorbed oxygen and re-injects the carrier, resulting in the reduction in the resistance of Ga-doped ZnO films. The possibility of reactions of ethanol gas with the surface of Ga-doped ZnO film can be explained as two oxidation states, where [O] represents the surface oxygen ions^34^.

3 $${\text{C}}_{{2}} {\text{H}}_{{5}} {\text{OH}}\,\left( {\text{g}} \right) + \left[ {\text{O}} \right] \to {\text{CH}}_{{3}} {\text{CHO}} + {\text{H}}_{{2}} {\text{O}}\,\left( {{\text{the}}\,{\text{dehydrogenation}}\,{\text{to}}\,{\text{acetaldehyde}}} \right)$$

4 $${\text{C}}_{{2}} {\text{H}}_{{5}} {\text{OH}}\,\left( {\text{g}} \right) + \left[ {\text{O}} \right] \to {\text{C}}_{{2}} {\text{H}}_{{4}} + {\text{H}}_{{2}} {\text{O}}\,\left( {{\text{the}}\,{\text{dehydration}}\,{\text{to}}\,{\text{ethylene}}} \right)$$

The first reaction represents the initiation of oxidation by dehydrogenation to CH_3_CHO. The dehydration to C_2_H_4_ initiates the second reaction. However, the selectivity for above mentioned reactions is initiated by acid–base properties of the oxide surface. The dehydration takes place on the acid surface, while the dehydrogenation process is on the basic surface^34^. The ethylene and acetaldehyde are subsequently reduced to CO_2_ and H_2_O. The depletion region due to the chemisorption of oxygen on film surface extends deeply at higher temperature. This provides scope for more gaseous elements to be adsorbed, resulting in the better sensing response. The hydroxyl group also desorbs at higher temperatures^35^. Hence at lower temperature (< 150 °C), the sensor surface is not desorbed completely and thus cause a smaller change in the resistance. The response of ethanol vapous was found to be promoted effectively by Ga-doped ZnO thin film.

### Sensing mechanism of acetone with Ga-doped thin film

The sensing mechanism of Ga-doped ZnO thin film to acetone may be described as follows. As the film is heated in air, the oxygen is adsorbed on zinc oxide surface and the surface reactions proceed slowly at lower temperature. Th ionic species such as O^2−^, O_2_^−^ and O^−^ are formed due to adsorption of oxygen. These species acquire electrons from the conduction band and desorbed from the surface at 80, 130 and 500 °C. The reaction kinematics is as follows^36^:

5 $${\text{O}}_{{2}} \,\left( {{\text{gas}}} \right) < = > {\text{O}}_{{2}} \,\left( {{\text{absorbed}}} \right)$$

6 $${\text{O}}_{{2}} \,\left( {{\text{absorbed}}} \right) + {\text{e}}^{ - } < = > {\text{O}}_{2}^{ - }$$

7 $${\text{O}}_{2}^{ - } + {\text{e}}^{ - } { < = > }2{\text{O}}^{ - }$$

The acetone may react with ionic oxygen species by two different ways with different rate constants (*k*), depending upon the temperature^37^:

$$\begin{aligned} {\text{CH}}_{{3}} {\text{COCH}}_{{3}} \,\left( {{\text{gas}}} \right) + {\text{O}}^{ - } & \to {\text{CH}}_{{3}} {\text{COCH}}_{2} + {\text{OH}}^{ - } + {\text{e}}^{ - } \\ & \quad k = 1.0 \times 10^{12} \,\exp ( - 21000/{\text{RT}})\,\left[ {{\text{cm}}^{3} /{\text{mol}}\,{\text{s}}} \right] \\ {\text{CH}}_{{3}} {\text{COCH}}_{{3}} \,\left( {{\text{gas}}} \right) + {\text{OH}}^{ - } & \to {\text{CH}}_{{3}} {\text{CHO}} + {\text{CH}}_{{3}} {\text{O}}^{ - } \\ & \quad k = 2.0 \times 10^{12} \,\exp ( - 63000/{\text{RT}})\,\left[ {{\text{cm}}^{{3}} {\text{/mol}}\,{\text{s}}} \right] \\ {\text{CH}}_{{3}} {\text{CHO}} + {\text{O}}\,\left( {{\text{bulk}}} \right) & \to {\text{CH}}_{{3}} {\text{COOH}} + {\text{O}}\,\left( {{\text{vacancies}}} \right) \\ {\text{CH}}_{{3}} {\text{COCH}}_{{3}} \,\left( {{\text{gas}}} \right) + {\text{O}}^{ - } & \to {\text{CH}}_{{3}} {\text{CO}} + {\text{CH}}_{{3}} {\text{O}}^{ - } + {\text{e}}^{ - } \\ & \quad k = 1.0 \times 10^{12} \,\exp ( - 42000/RT)\,\left[ {{\text{cm}}^{{3}} {\text{/mol}}\,{\text{s}}} \right] \\ {\text{CH}}_{{3}} {\text{C}}^{ + } {\text{O}} & \to {\text{C}}^{ + } {\text{H}}_{{3}} + {\text{CO}} \\ & \quad k = 2.0 \times 10^{11} \times \exp \,( - 15000/RT)\,\left[ {1/{\text{s}}} \right] \\ {\text{CO}} + {\text{O}}^{ - } & \to {\text{CO}}_{{2}} + {\text{e}}^{ - } \\ \end{aligned}$$ (8)

## Results and discussion

In the present investigation, the gas sensing property of Ga-doped ZnO thin films modified by swift heavy irradiation technique have been studied. The Ga-doped ZnO thin films were irradiated by 120 MeV Ag^9+^ and 70 MeV Si^6+^ ion beams of different fluences. The fabricated thin films of 5at.% ZnO:Ga sensors were irradiated with 120 MeV Ag^9+^ ion fluence while 3at.% ZnO:Ga films were irradiated by 70 MeV Si^6+^ ion beam. The films were irradiated by 1 × 10^12^, 2 × 10^13^ and 5 × 10^13^ ions/cm^2^ fluences of Ag^9+^ and Si^6+^ ion beams. The XRD patterns of Ag^9+^ ion irradiated 5at% Ga doped ZnO thin film show all peaks corresponding to the hexagonal wurtzite structure with polycrystalline structure. It is observed that the intensity of all peaks increases with increasing ion fluence. This shows that irradiation enhances the crystallization of Ga-doped ZnO films. The modified Ga-doped ZnO thin films were exposed to different concentrations of ethanol and acetone for gas sensing applications.

### X-ray photoelectron spectroscopy (XPS) of 3at.% ZnO:Ga thin films

**Figure 2:**
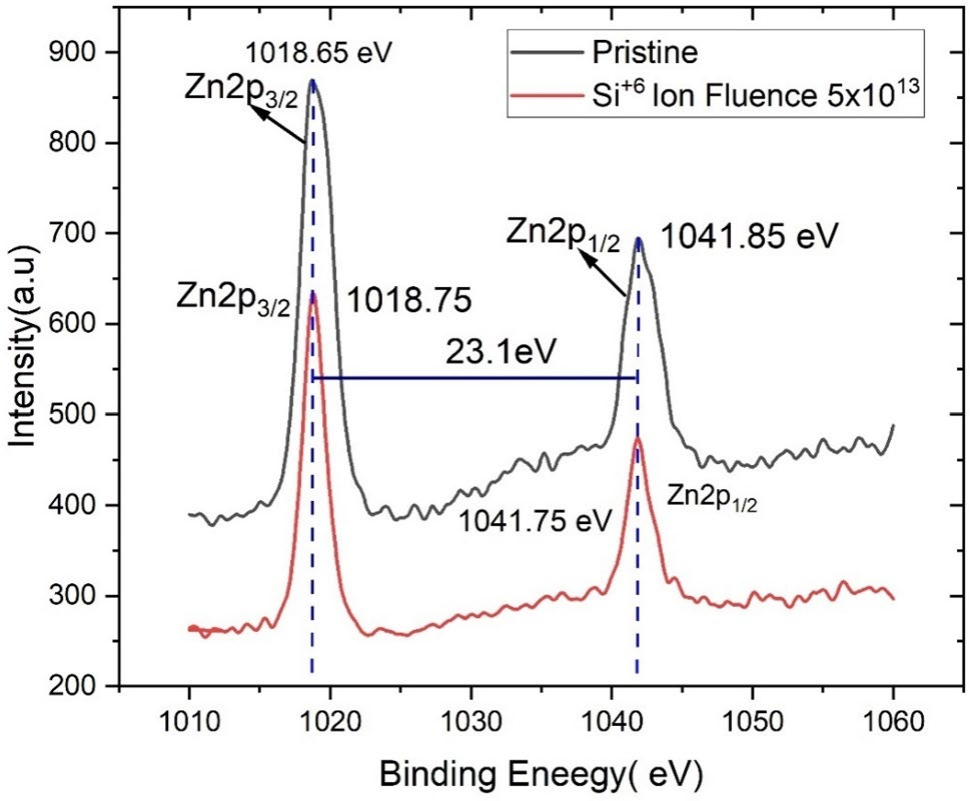
XPS spectra related to Zn binding energies for the pristine and Si^6+^ ion beam irradiated ZnO:Ga thin film.

The chemical states of gallium, zinc, and oxygen were identified by obtaining the XPS spectra of the 3% Ga doped thin film (Pristine) and 3% Ga doped thin film irradiated by Si^6+^ ion fluence at 5 × 10^13^ ions/cm^2^. Figure 2 depicts the wide energy range (0–1070 eV) XPS spectra for both films and demonstrate the distinct splitting of the Zn 2p spectral line into the 2p_1/2_ and 2p_3/2_ core levels.

**Figure 3:**
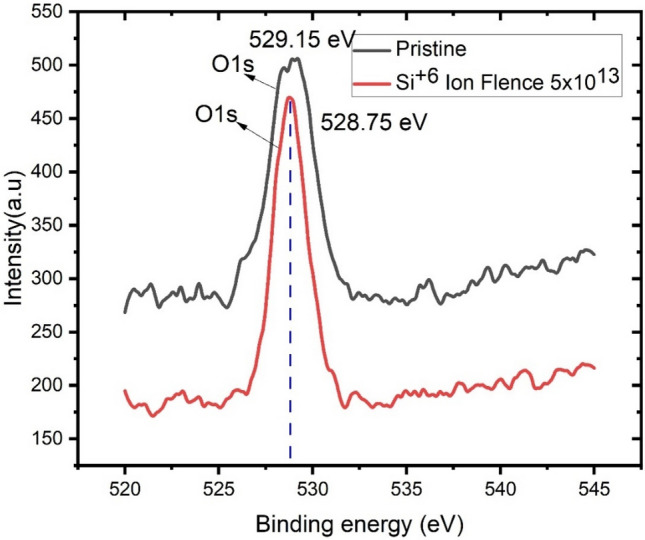
XPS spectra of O1s peak for the pristine and Si^6+^ ion beam irradiated ZnO:Ga thin film.

For 3% Ga doped pristine thin film, doublet lines of Zn analogous to 2p_3/2_ and 2p_1/2_ are observed at 1018.65 and 1041.85 eV, respectively. For a 3% Ga doped thin film irradiated by Si^6+^ ion fluence at 5 × 10^13^ fluence, the 2p_3/2_ and 2p_1/2_ core levels of Zn2p were observed at 1018.75 eV and 1041.75 eV, respectively. This verifies that a significant number of Zn atoms remains in the film with the same formal valence state of Zn^2+^ within an oxygen-deficient ZnO matrix. The binding energy difference between Zn2p^3/2^ and Zn2p^1/2^ emissions was found to be 23.1 eV, which is the characteristic value for ZnO^38^. The O1s XPS spectra (Fig. 3) for both Pristine and irradiated thin films were observed at 529.15 and 528.75 eV, which correspond to O^2−^ ions at the intrinsic sites on the hexagonal Zn^2+^ wurtzite structure^39^.

**Figure 4:**
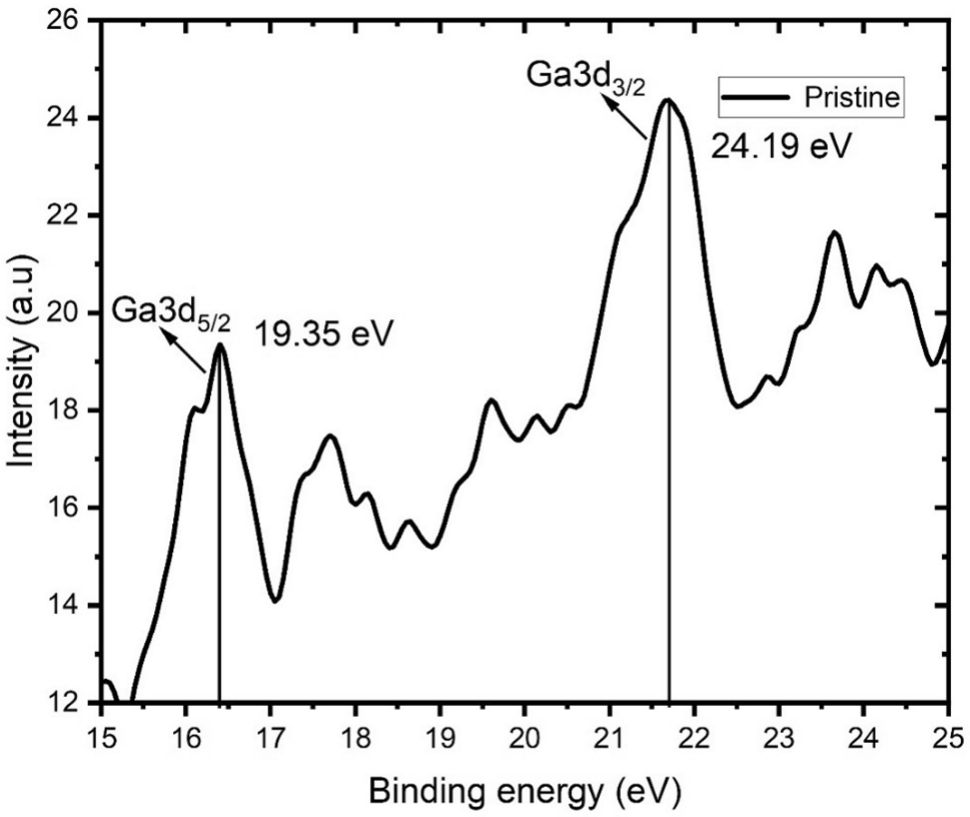
XPS spectra related to Ga binding energies for the pristine ZnO:Ga thin film.

**Figure 5:**
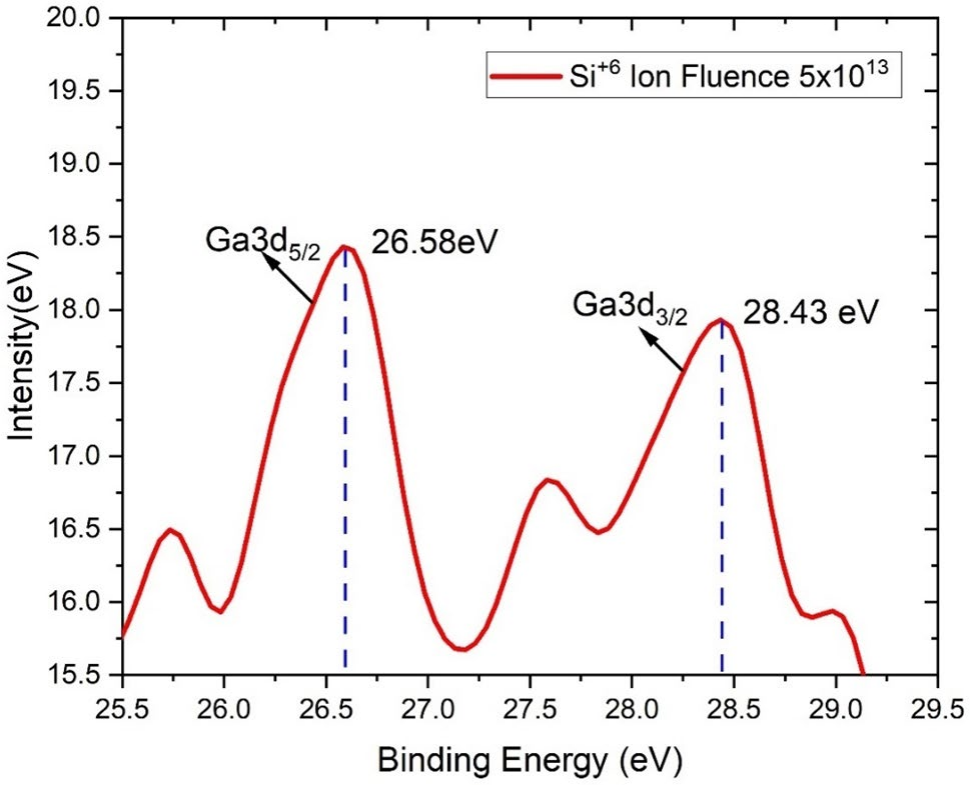
XPS spectra related to Ga binding energies for the Si^6+^ ion beam irradiated ZnO:Ga thin film.

The XPS data of the Ga-doped ZnO thin films are shown in Figs. 4 and 5. The binding energies of the Ga3d_5/2_ and Ga3d_3/2_ spectra were observed at 19.35 eV and 24.19 eV (Fig. 4). The spectra clearly show the presence of Ga in the doped materials.

Binding energy of Ga3d_5/2_ and Ga3d_3/2_ spectra after irradiation with Si^6+^ ions beams were observed at 26.58 eV to 28.43 eV (Fig. 5). This indicated that after ion beam irradiation, the ZnO:Ga films register a shift towards higher energies. This shift in binding energy may be attributed to a change in the resistivity of the ZnO:Ga film after irradiation and can be interpreted as a Burstein-Moss effect (Correia et al., 2018). Higher ionic bombardment, on the other hand, reduces the substitutional doping of Ga in the ZnO lattice due to the level of structural atomic disorder and re-evaporation of Zn, resulting in an increase in the electrical resistivity.

### Structural analysis of ZnO:Ga thin films (3at.%, 5at.%)

**Figure 6:**
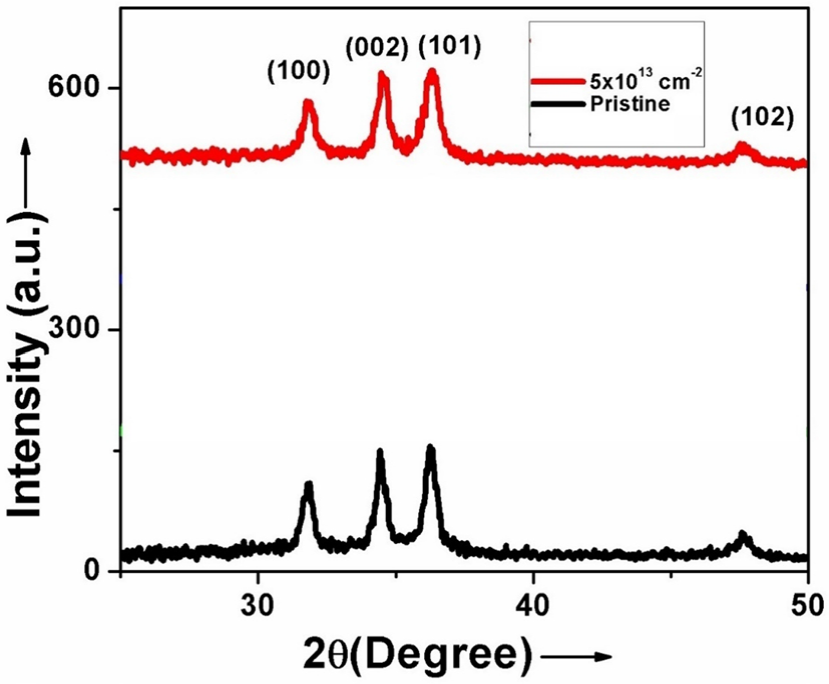
XRD patterns of pristine and 120 MeV Ag^9+^ ions beam irradiated 5at.% ZnO:Ga thin films.

**Figure 7:**
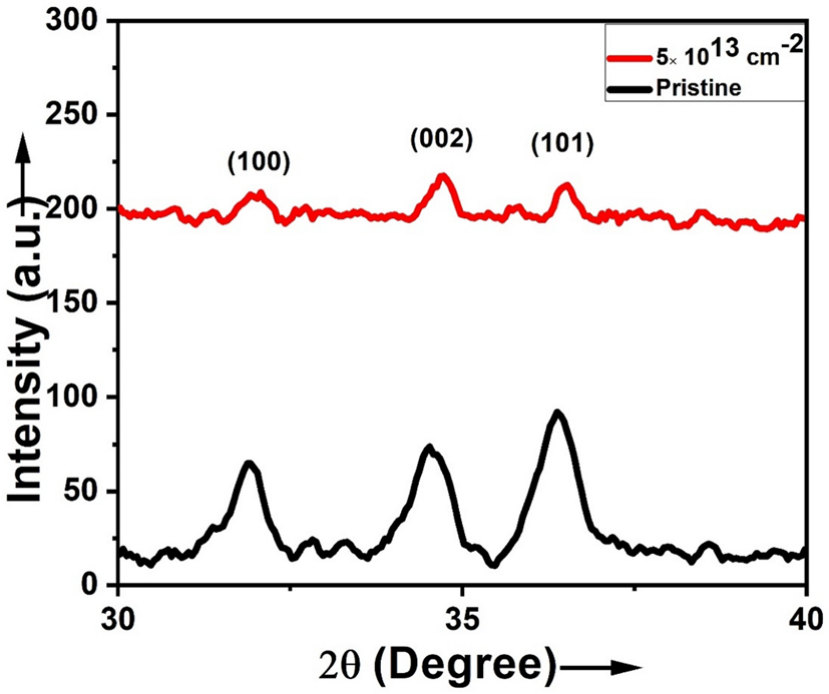
XRD patterns of pristine and 70 MeV Si^6+^ ions beam irradiate 3at.% ZnO:Ga thin films.

The GIXRD pattern of the pristine, silver beam irradiated 5at.% ZnO:Ga thin films and silicon beam irradiated 3at.% ZnO:Ga thin films, deposited on silicon substrate, are shown in Figs. 6 and 7, respectively. The figures illustrate diffraction peaks from the (100), (002), (101), and (102) planes. A prominent diffraction peak (101) of hexagonal wurtzite-structured ZnO was observed in the patterns of both pristine and irradiated films.

In order to observe the change in the crystallite size of films induced by SHI irradiation, the average crystallite size was calculated using Scherrer’s formula (Scherrer, 1918).

9 $${\mathcal{D}} = \frac{0.9\lambda }{{\beta \cos \theta }}$$

where *β* is the full width at half maximum (FWHM) in radian, λ is wavelength of X-ray (1.5406 Å for Cu-K_α_) and θ is Bragg angle. Average crystallite size and lattice parameters of ZnO:Ga thin films irradiated with silver and silicon beams, determined using diffraction pattern are summarized in Table 1.

**Table 1:** Average crystallite size and lattice parameters of 5at.% ZnO:Ga irradiated by Ag^9+^ ions and 3at.% ZnO:Ga irradiated by Si^6+^ ions beams.

Sample	Ion beam	Fluence (ions/cm^2^)	Average crystallite size (nm)	Lattice constant ‘a’(Å)	Lattice constant ‘c’(Å)	Stress (Pa)	Strain
5at.% ZnO:Ga	Ag^9+^	Pristine	18	3.245	5.198	+ 8.95 × 10^−8^	-0.0015
		5 × 10^13^	14	3.224	5.190	+ 9.18 × 10^−8^	-0.0030
3at.% ZnO:Ga	Si^6+^	Pristine	36	3.228	5.190	+ 9.18 × 10^−8^	-0.0030
		5 × 10^13^	14	3.226	5.164	+ 2.40 × 10^−8^	-0.0080

When irradiated by Ag^9+^ and Si^6+^ ion beams with fluences of 5 × 10^13^ ions/cm^2^, the crystallite size decreases from 18 to 14 nm and 36 nm to 14 nm, respectively. It is because the charged ions of the cylindrical shock wave produced by SHI force the atoms out of their original position and shatter their lattices^40^. The FWHM of the dominant diffraction peak (101) increased, resulting in the observed decrease in crystallite size. The FWHM of a diffraction peak is affected by inhomogeneous strain and grain boundary defects in the crystallites^41^. With increasing fluences, the position of the (101) peak shifted slightly from 36.19° to 36.35° for Ag^9+^ ions irradiated films and 36.37° to 36.53° for Si^6+^ ions irradiated films. The lattice parameters ‘*a’* and ‘*c’* of films are determined from the diffraction peaks (002) and (101) using the relations for the hexagonal structure of ZnO (a = λ/√3sin *θ* and c = λ/sin*θ*), where *θ* is the wavelength of x-ray and *θ* is the angle of incidence^42^. Based on the values of lattice parameters, the stress in the films was also calculated using the following equation^43^:

10 $$\sigma = - 233 \times 10^{9} \left[ {\frac{{C - C_{0} }}{{C_{0} }}} \right]\,{\text{Pa}}$$

where *σ* is the stress, c is lattice parameter of the film and C_o_ is strain free lattice parameter, which is 0.5206 nm.

In the case of 5at.% ZnO:Ga thin films, it is found that pristine samples have a stress of tensile nature, which increases after irradiation by Ag^9+^ ions beam. In the case of 3at.% ZnO:Ga thin films, the stress is tensile in nature, which decreases after irradiation by Si^6+^ ions beam. Tensile stress is also present in 3at.% ZnO:Ga thin films, which decreases after irradiation by Si^6+^ ions beam. The energy transfer from SHI to the ZnO lattice caused the variation in stress. The stresses in thin films will deform the unit cell and introduce unwanted anisotropy, which has large effects on the structural, mechanical, electrical, and optical properties of the film^44^. Ungar et al.^45^ show that the excess volume of grain boundaries associated with vacancies and vacancy clusters is the source of lattice strain to the stress field. The XRD pattern could be used to assess the mean magnitude of the local lattice strains in a nanocrystalline material^46^. It is observed that strain in film increases after irradiation by ion beams.

### Scanning electron microscopy

**Figure 8:**
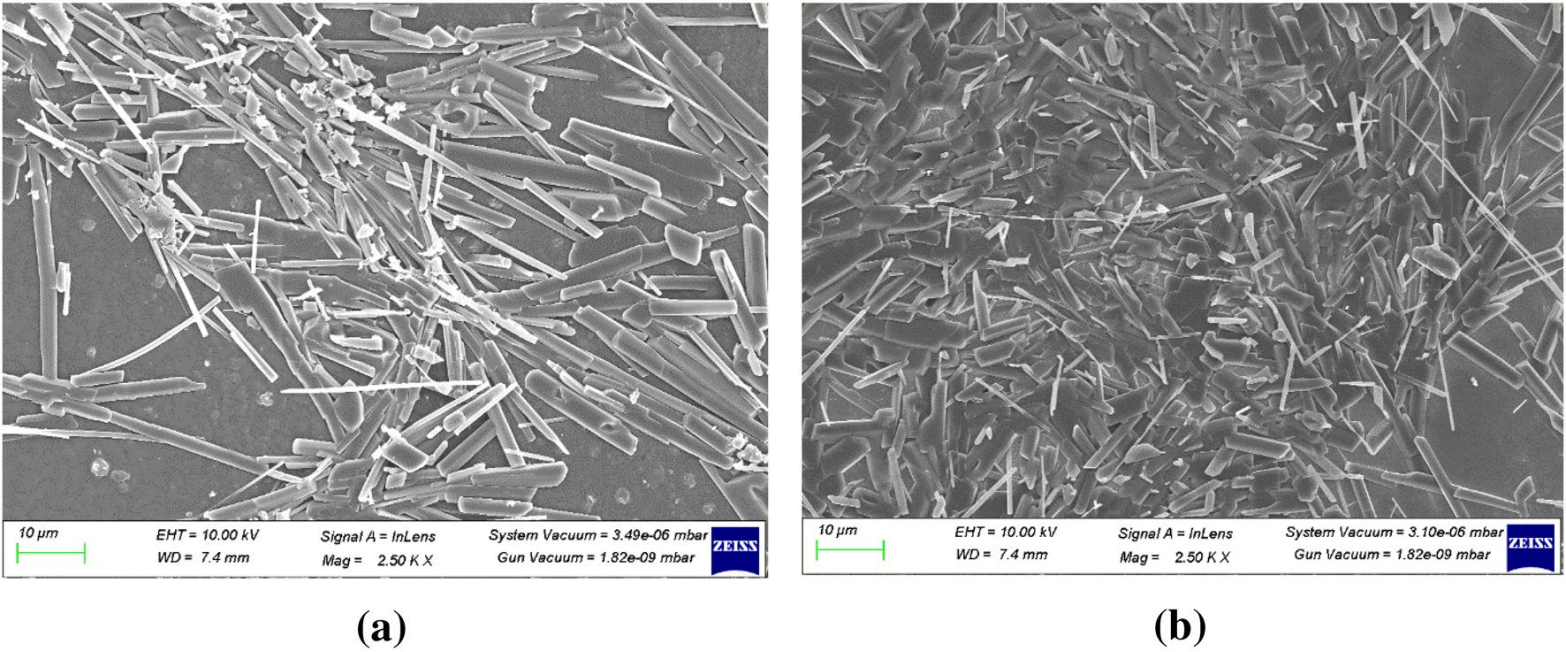
SEM structures of 5at.% ZnO:Ga thin films for (**a**) pristine and (**b**) irradiation with Ag^9+^ ion beam.

**Figure 9:**
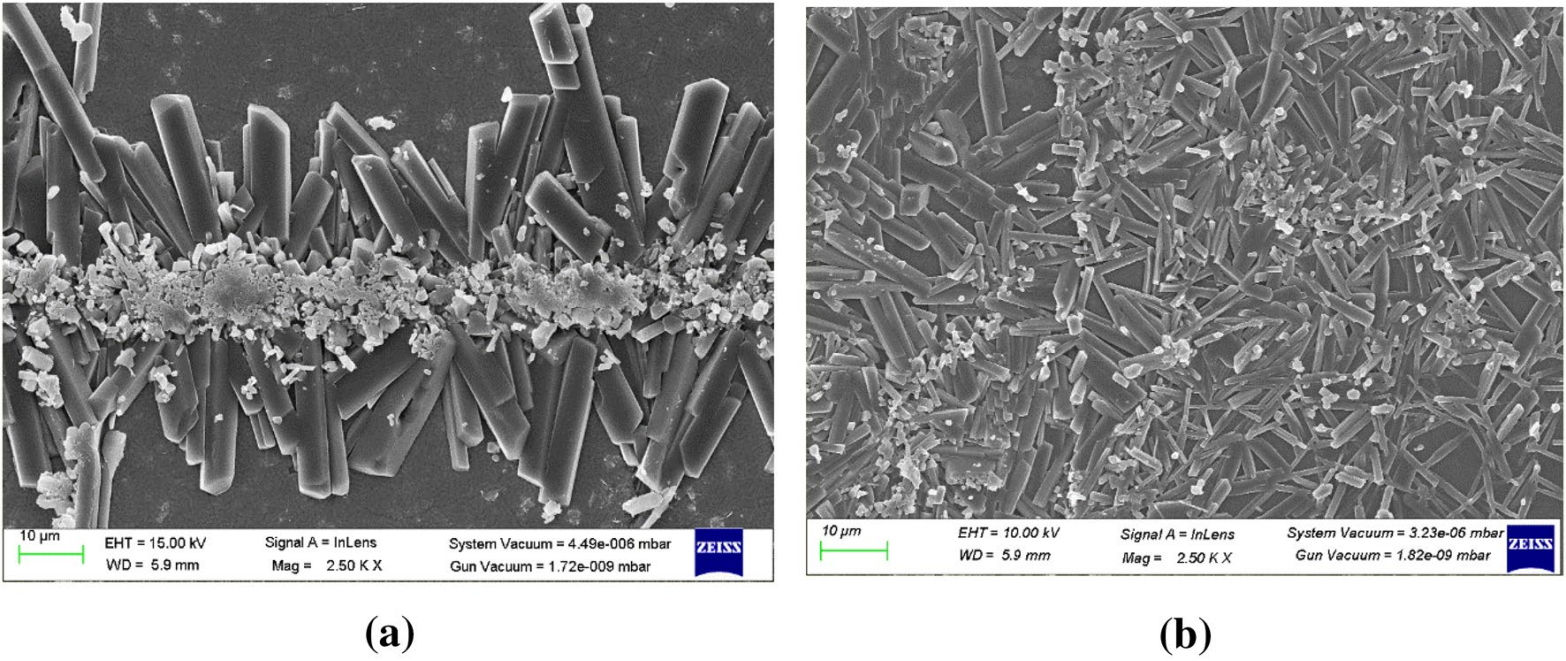
SEM structures of 3at.% ZnO:Ga thin films for (**a**) pristine and (**b**) irradiation with Si^6+^ ion beam of the fluences 5 × 10^13^ ions/cm^2^.

The surface morphological studies of 5at.% ZnO:Ga and 3at.% ZnO:Ga thin films carried out by taking SEM images before and after irradiation by Ag^9+^ and Si^6+^ ion beams with fluences of 5 × 10^13^ ions/cm^2^ are presented in Figs. 8 and 9 Crystal-like structures were observed in the unexposed Ga-doped thin films. After irradiation with Ag^9+^ and Si^6+^ the size of crystals decreases, which supports the data reported in Table 1 utilising XRD measurements.

### Gas sensing response of 5at% Ga-doped ZnO thin film irradiated by 120 MeV Ag^9+^ ion beam

**Figure 10:**
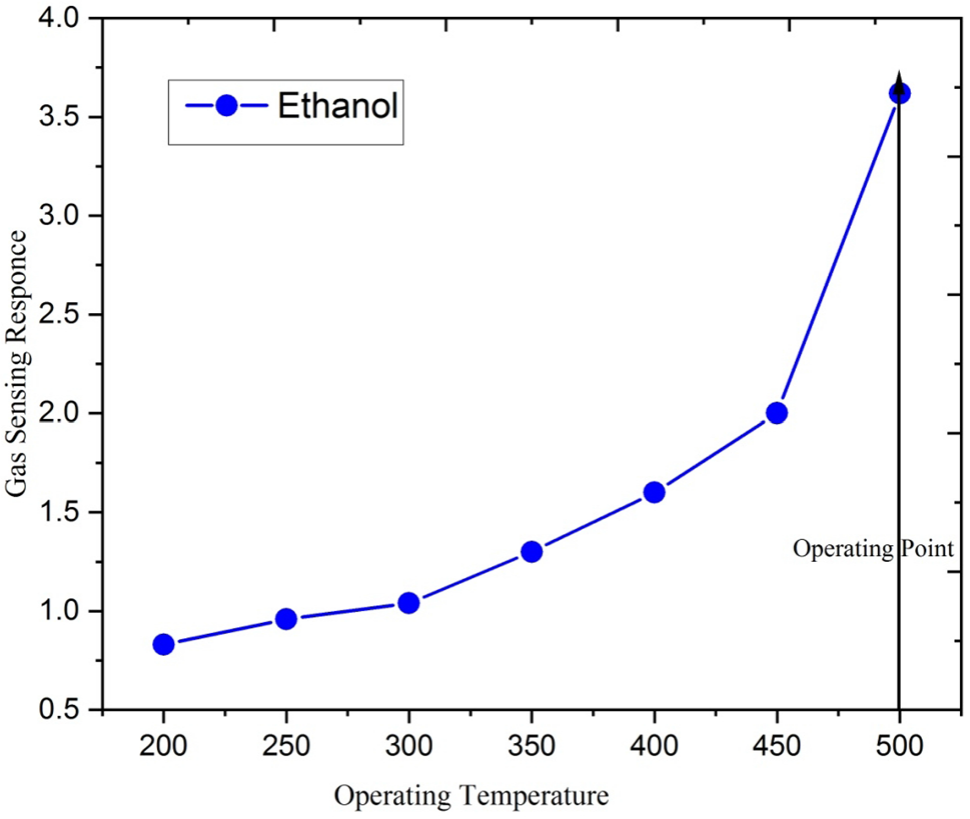
Response of the 5at% Ga-doped ZnO thin film (Ag^9+^ ion irradiated at 5 × 10^13^ ion fluence) for 250 ppm ethanol concentration at different operating temperature.

The modification of Ga doped ZnO thin film after the ion irradiation is due to energy deposition in the ZnO lattice ion. Figure 10 represents the response of the 5at % Ga-doped ZnO thin film irradiated with Ag^9+^ ion at ion fluence of 5 × 10^13^ ions/cm^2^ to 250 ppm ethanol concentration at different operating temperatures.

The ethanol gas sensing response of the film remains almost constant between the operating temperature 200 °C and 300 °C. At low operating temperature region, the sensing response of Ga-doped ZnO is controlled by the speed of chemical reaction as the gas molecules do not possess sufficient thermal energy to react with surface-adsorbed oxygen species. The response of the film slowly increases with increasing temperature form of 300 to 450 °C. After 450 °C, the sensitivity increases rapidly up to the maximum operating temperature (500 °C) of the gas sensing setup. This increment in sensitivity is due to decrees in the resistance of Ga-doped ZnO film, which is probably due to a change in the inter-granular potential barrier and intrinsic resistance of crystallites. At high temperature, large numbers of gas molecules also get sufficient energy required for the chemical reaction.

The response is found to be maximum at the operating temperature of 500 °C for 250 ppm concentration of ethanol. The pristine sample (annealed at 700 °C) was optimized at 450 °C temperature for 250 ppm concentration of ethanol. After irradiation with 5 × 10^13^ ion/cm^2^, the optimum temperature has been shifted to 500 °C for same concentration of ethanol. This shift may be due to material modification after ion beam irradiation. After irradiation, the surface-to-volume ratio of nanocrystalline film increases and thus allowing a greater number of gas molecules for reaction with adsorbed oxygen.

**Figure 11:**
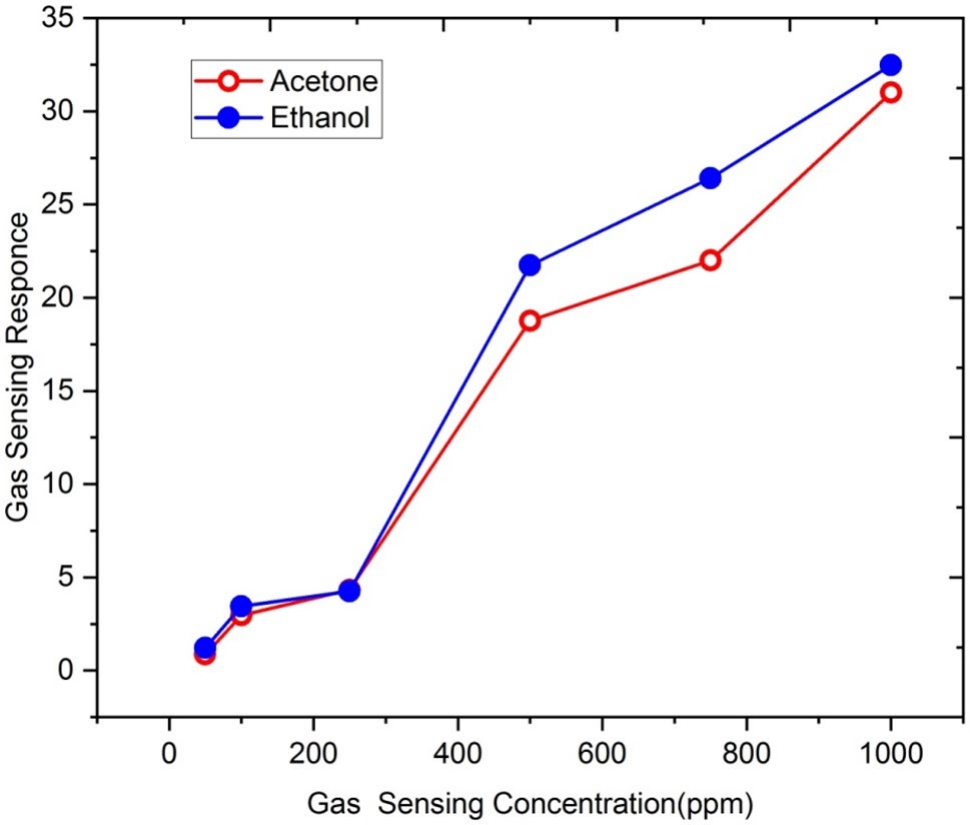
Sensing response of 5at% Ga-doped ZnO thin film (Ag^9+^ ion irradiated with 5 × 10^13^ ion/cm^2^ fluence) for ethanol and aceton (250 ppm concentration) at 500 °C operating temperature.

Figure 11 represents the sensing response of 5at% Gallium doped ZnO thin film irradiated with 5 × 10^13^ ions/cm^2^ fluence of Ag^9+^ ion for different concentrations of ethanol and acetone gases ranging from 50 to 1000 ppm.

At the operating temperature 500 °C, the response of thin film for ethanol is slow between 50 to 250 ppm concentration. The low sensing response of thin film may be due to deficiency of ethanol gas molecules to interact with adsorbed oxygen species like O_2_^−^, O^2−^ and O^−^. The gas sensing response increases rapidly for gas concentration between 250 and 500 ppm and thereafter it increases linearly.

This increase in gas sensing response with gas concentration may be due to the sufficient availability of adsorbed oxygen species on the film surface. However, after 750 ppm, there is a gradual increase in sensor response. This is because the thin surface begins to attain saturation in respect of gas reactivity. Initially, the molecular Oxygen was adsorbed on Ga-doped ZnO film surface and electrons were consumed after the reactions, and with oxygen adsorption at the thin film surface its resistance increases.

11 $$\begin{gathered} {\text{O}}_{{2}} \,\left( {{\text{gas}}} \right) \leftrightarrow {\text{O}}_{{2}} \,\left( {{\text{adsorbed}}} \right) \hfill \\ {\text{O}}_{{2}} \,\left( {{\text{adsorbed}}} \right) + {\text{e}}^{ - } \leftrightarrow {\text{O}}_{{2}}^{ - } \,\left( {{\text{adsorbed}}} \right) \hfill \\ {\text{O}}_{2}^{ - } \,\left( {{\text{adsorbed}}} \right) + {\text{e}}^{ - } \leftrightarrow 2{\text{O}}^{ - } \,\left( {{\text{adsorbed}}} \right) \hfill \\ \end{gathered}$$

When Ga-doped ZnO thin film sensor is exposed to ethanol vapour, the chemisorbed oxygen ions react with its atoms, produce CO_2_ and H_2_O molecules and release electrons after consuming chemisorbed oxygen from the surface of the film. The reaction can be written as:

12 $${\text{C}}_{{2}} {\text{H}}_{{5}} {\text{OH}} + 6{\text{O}}^{ - } \leftrightarrow 2{\text{CO}}_{2} + 3{\text{H}}_{{2}} {\text{O}} + 6{\text{e}}^{ - }$$

As a result, the resistance of the film decreases due to the release of electrons back into the conduction band.

Similar behavior is observed for acetone gas. The response of acetone gas sensing is very low up to 250 ppm but at higher concentrations, the sensing response increases very sharply. It is evident from the figure that not only the temperature, but the concentration levels of ethanol and acetone also play a role in determining the sensing response of the film.

**Figure 12:**
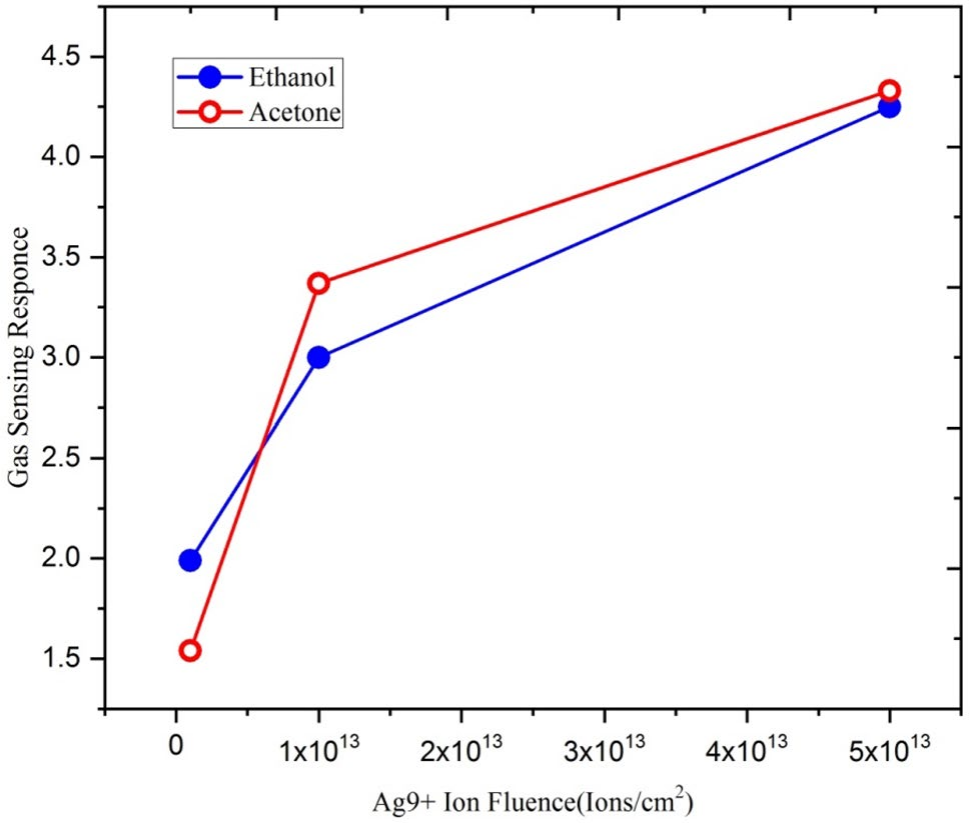
Variation of gas sensing response with different fluence of Ag^9+^ ion.

Figure 12 shows the variation of sensing response of Ga-doped ZnO thin film at 250 ppm concentration of ethanol and acetone at different fluence of Ag^9+^ (1 × 10^12^, 1 × 10^13^ and 5 × 10^13^) at optimizing operating temperature 500 °C. It is evident from the figure that gas sensing response for both ethanol and acetone gas vapours increases with increasing the ion fluence.

Table 2 shows the variation of crystallite size of Ga doped ZnO thin film with silver ion fluence.

**Table 2:** XRD results showing the variation in crystallite size with Ag^9+^ ion beam fluence.

S. no	Ag^9+^ Ion fluence (ions/cm^2^)	Crystallite size (nm)
1	1 × 10^12^	26
2	1 × 10^13^	15
3	5 × 10^13^	14

**Figure 13:**
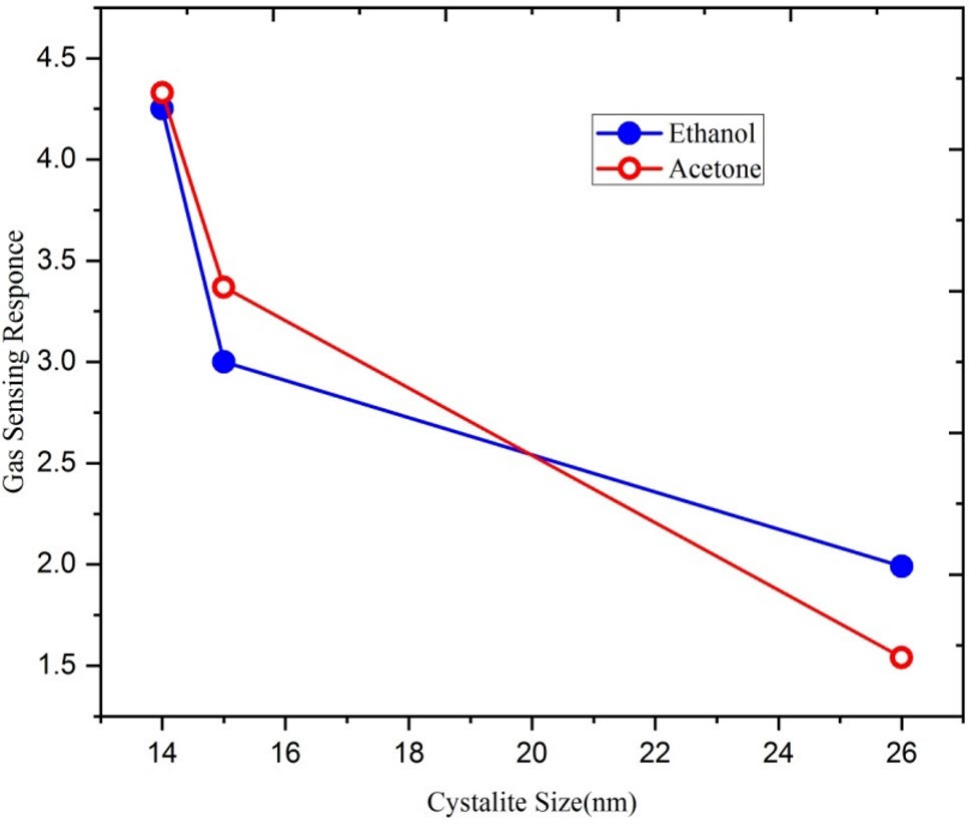
Variation of ethanol and acetone gas sensing response with crystallite size.

From the XRD results, it is evident that the crystallite size decreases with increasing ion fluences. This increase is due to an increase in surface-to-volume ratio in nanocrystalline films. As the crystallite size is reduced, the grain boundary contacts exhibit higher resistance and govern the electric gas sensitivity. Swift heavy ions have improved the sensitivity of Ga-doped ZnO by reducing the particle size. It is evident from Fig. 13 that the gas sensing response for both ethanol and acetone gases increases with a decrease in crystallite size.

**Figure 14:**
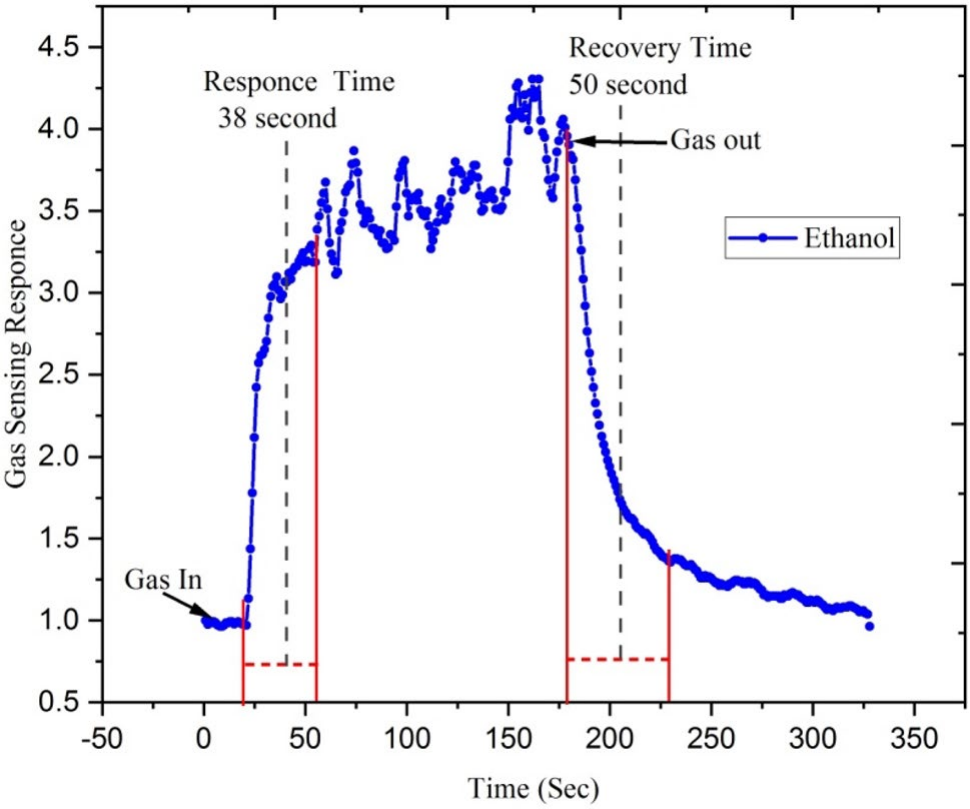
Response-recovery characteristics of the Ga-doped ZnO (Ag^9+^ ion irradiated at 5 × 10^13^ ion fluence) thin film at the operating temperature 500 °C with 250 ppm concentration of ethanol.

**Figure 15:**
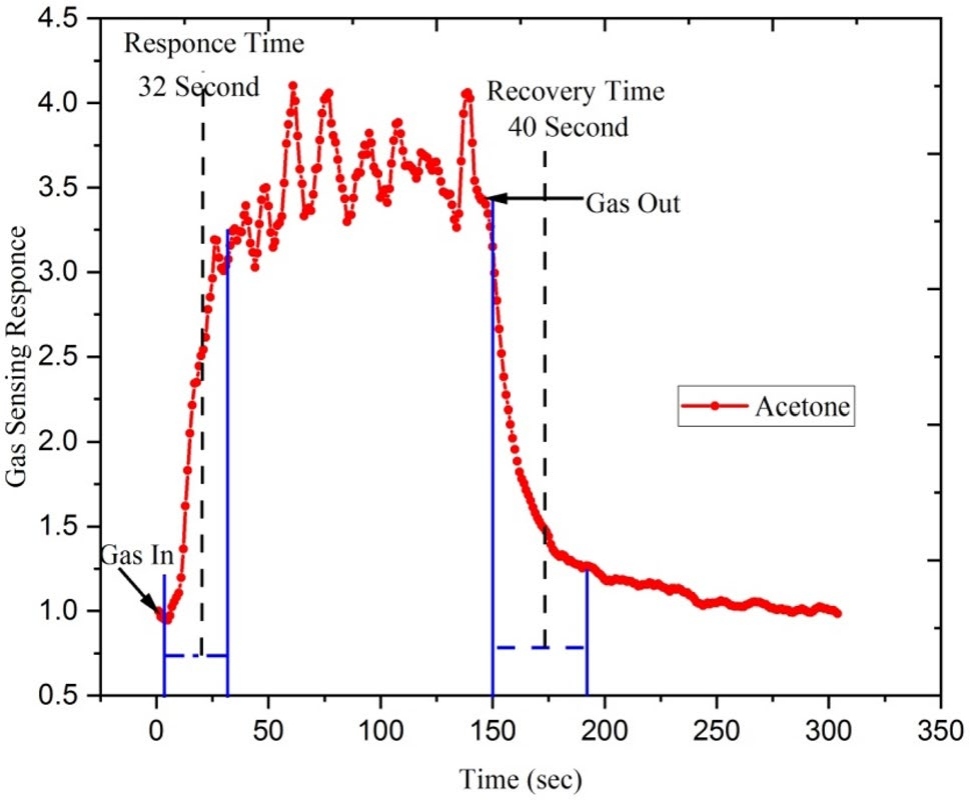
Response-recovery characteristics of the Ga doped ZnO (Ag^9+^ ion irradiated at 5 × 10^13^ ion fluence) thin film at the operating temperature 500 °C with 250 ppm concentration of acetone.

Figures 14 and 15 show response-recovery characteristics of the Ga doped ZnO thin film irradiated with Ag^9+^ ion irradiated at 5 × 10^13^ ion fluence at the operating temperature 500 °C with 250 ppm concentration of ethanol and acetone. Figure 14 shows that the response and recovery time of Ga doped ZnO thin film irradiated with Ag^9+^ exposed with ethanol is ~ 38 and ~ 50 s and Fig. 15 shows that with exposure to acetone gas this film has response and recovery time of ~ 32 and ~ 40 s. It means that silver beam irradiated thin films have a fast response to acetone gas.

**Figure 16:**
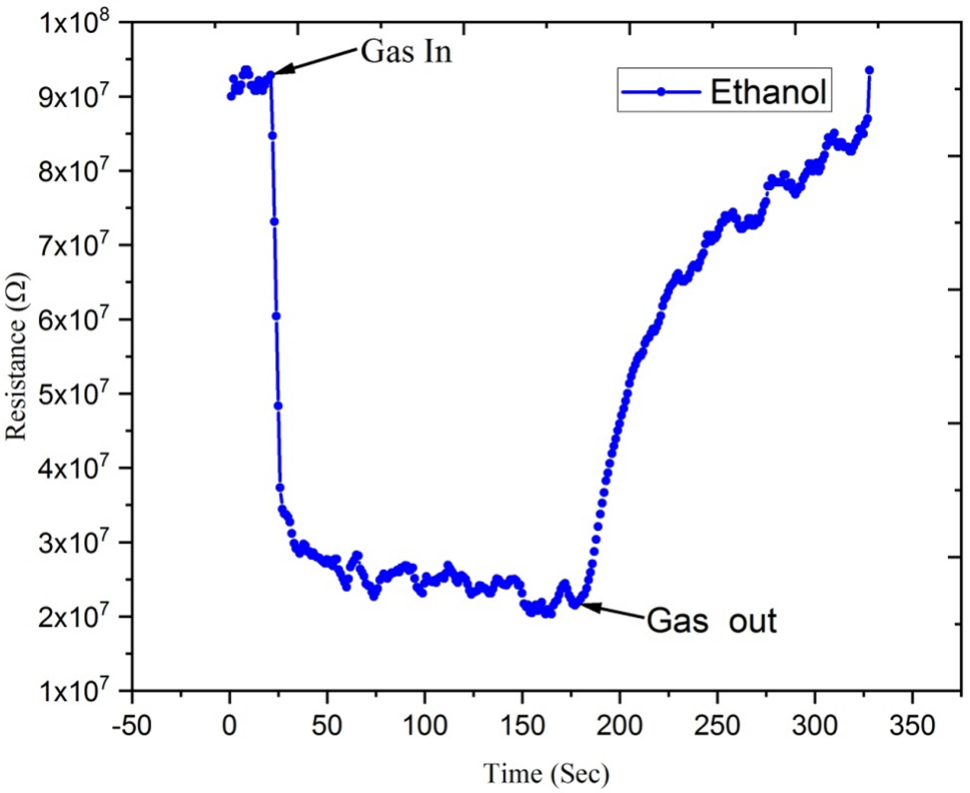
Variation of electrical resistance of Ga-doped ZnO thin film (Ag^9+^ ion irradiated at 5 × 10^13^ ion fluence) with time for 250 ppm concentration of ethanol gas.

**Figure 17:**
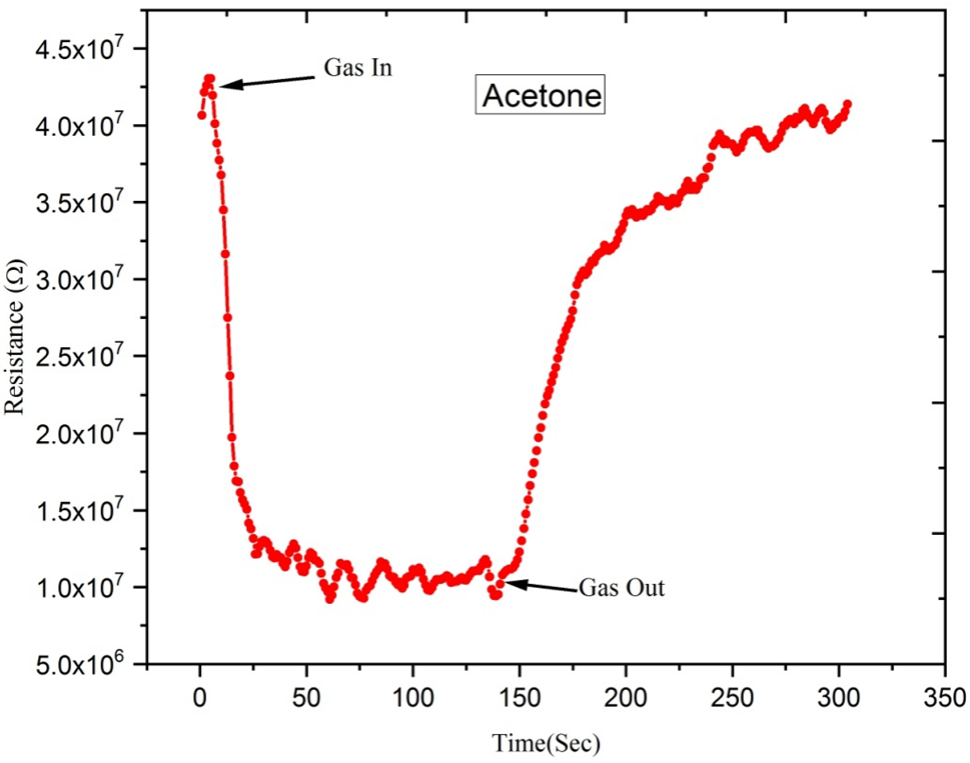
Variation of electrical resistance of Ga-doped ZnO thin film (Ag^9+^ ion irradiated at 5 × 10^13^ ion fluence) with time for acetone gas of 250 ppm concentration.

Figures 16 and 17 show variations in electrical resistance of Ga-doped ZnO thin film irradiated with Ag^9+^ ion at 5 × 10^13^ ion fluence with time for ethanol and acetone gas concentrations of 250 ppm.

These results show that with exposure to ethanol gas of 250 ppm concentration, the electrical resistance of Ga-doped ZnO thin film decreases from 9 × 10^7^ to 2 × 10^7^ Ω, while with exposure to acetone gas the electrical resistance of thin film decreases from 4.25 × 10^7^ to 1 × 10^7^Ω. This large variation of electrical resistance of Ga-doped ZnO thin film is due to the fact that when ethanol or acetone gas is injected in the chamber, it reacts with the adsorbed oxygen and releases the electron back to the conduction band, resulting in the decrease in the resistance. From the selectivity point of view from the result, it is evident that the response of the sensor is much higher for acetone in comparison to ethanol.

### Gas sensing response of 3at% Ga-doped ZnO thin film irradiated by 70 MeV Si^6+^ ion beam

**Figure 18:**
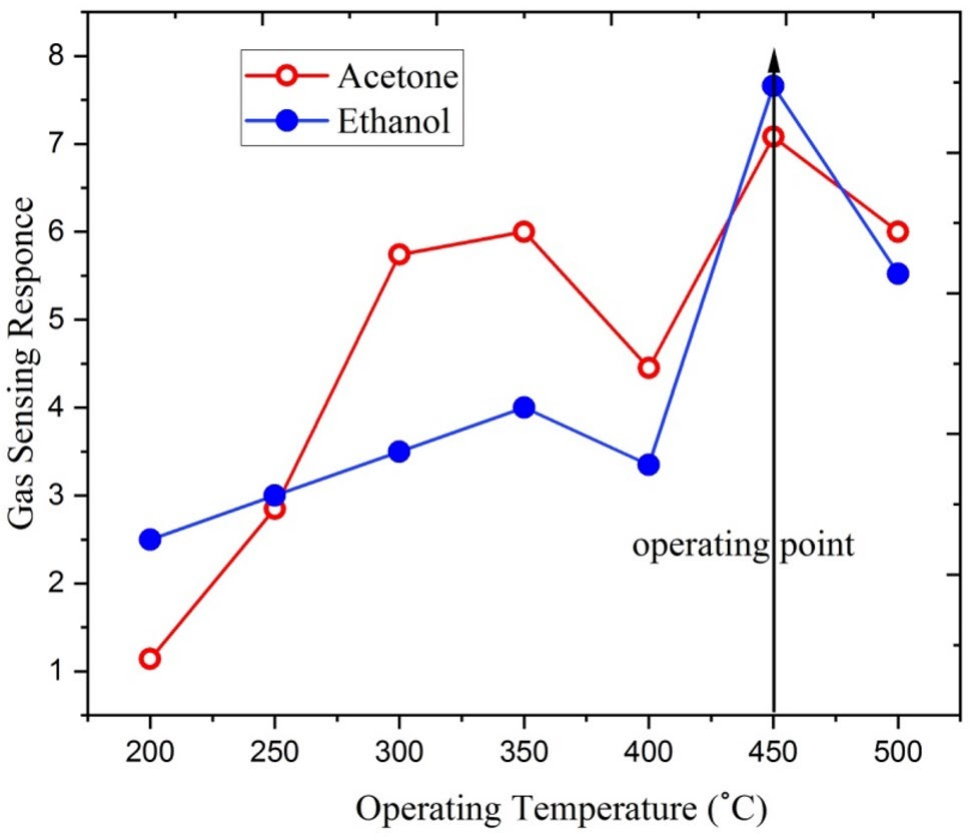
Response of the 3at% Ga-doped ZnO thin film (Si^6+^ ion irradiated at 5 × 10^13^ ion fluence) to 250 ppm ethanol and acetone concentrations at different operating temperature.

In the present investigation, 3at% gallium doped ZnO thin film was irradiated by 70 MeV Si^6+^ ions at different fluence (1 × 10^11^, 1 × 10^12^ and 5 × 10^13^) for gas sensing applications. Figure 18 shows the sensing response of 3at% Ga doped ZnO thin film irradiated with 70 MeV Si^6+^ ions at 5 × 10^13^ fluence for 250 ppm concentration of ethanol and acetone at different operating temperatures.

The sensing response of this thin film increases up to 350 °C and then decreases slightly between the temperatures 350 and 400 °C. Further, the thin film shows maximum sensing response at the temperature of 450 °C. This increase may be due to the presence of a large number of gas molecules for reaction with adsorbed oxygen species. The gas sensing response decreases on increasing the temperature beyond 450 °C. Hence the 3at% Ga doped ZnO irradiated with 5 × 10^13^ ion fluence of Si^6+^ exposed at 250 ppm concentrations of ethanol and acetone is optimized at 450 °C temperature. The optimization temperature of the thin film remains invariant i.e. 450 °C.

**Figure 19:**
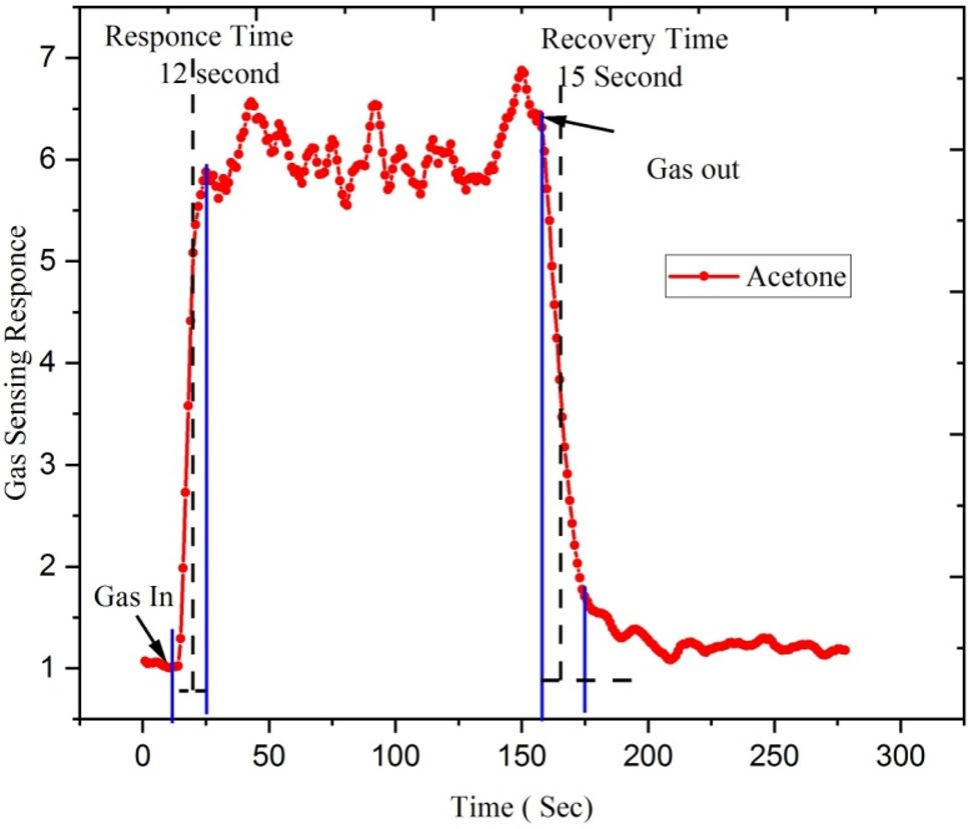
Response-recovery characteristics of the Ga-doped ZnO (Si^6+^ ion irradiated at 5 × 10^13^ ion fluence) thin film at the operating temperature 450 °C for 250 ppm concentration of acetone.

**Figure 20:**
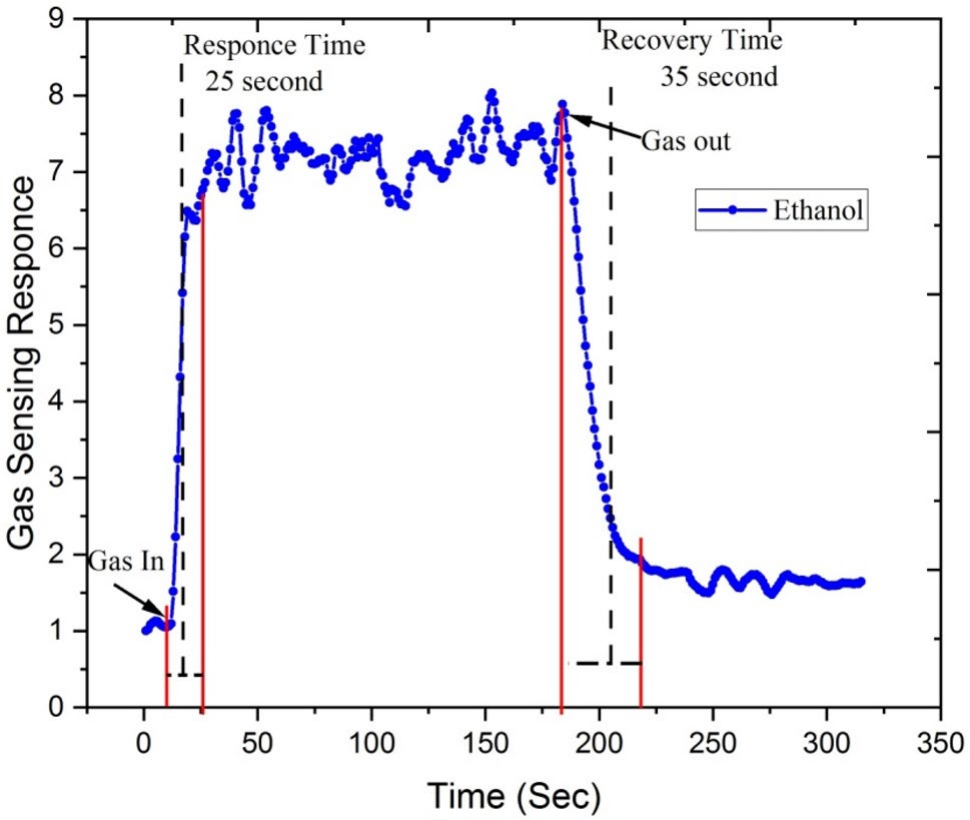
Response-recovery characteristics of the Ga-doped ZnO (Si^6+^ ion irradiated at 5 × 10^13^ ion fluence) thin film at the operating temperature 450 °C with 250 ppm concentration of ethanol.

Figures 19 and 20 show response-recovery characteristics of the Ga doped ZnO thin film irradiated with 5 × 10^13^ ion fluence of Si^6+^ ion at the operating temperature of 450 °C with 250 ppm concentration of acetone and ethanol.

The irradiated Ga doped ZnO thin film responds very rapidly after the introduction of ethanol and acetone gases and gets recovers when it is exposed to the air. The Si^6+^ ion irradiated Ga doped ZnO based sensor shows a response and recovery time of ~ 12 and ~ 15 s with the exposure of acetone gas of 250 ppm while it shows ~ 25 and ~ 35 s response and recovery time with the exposure of ethanol gas. It means that silicon beam irradiated thin films have a fast response to acetone gas.

**Figure 21:**
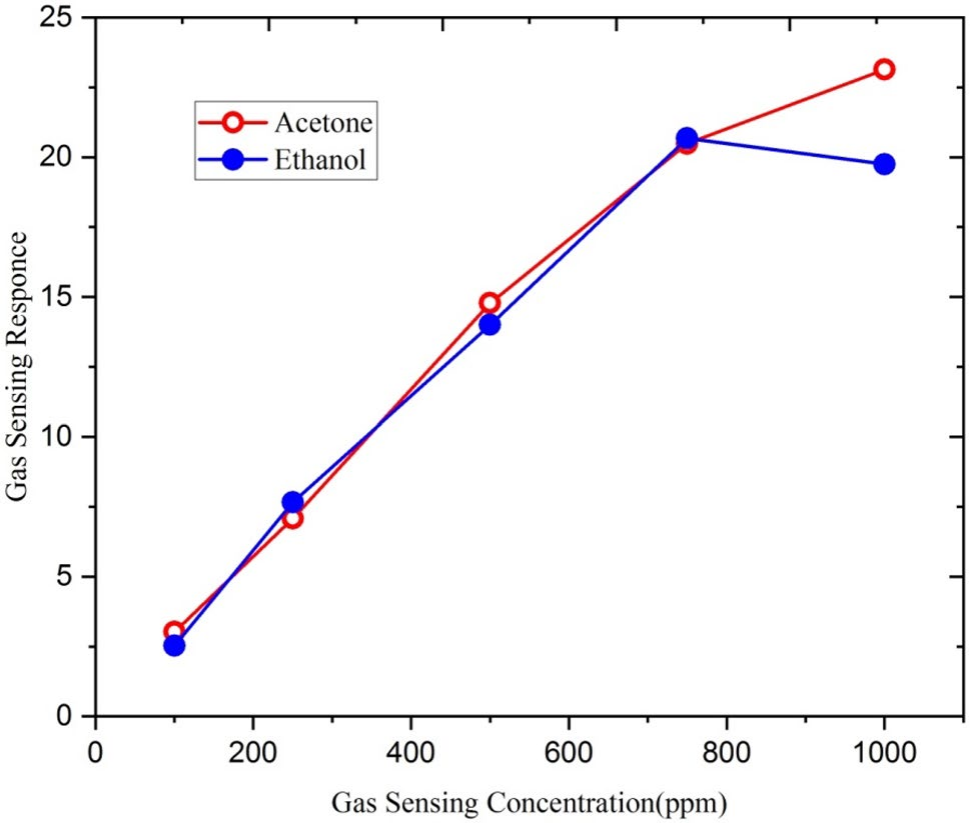
Sensing response of 3at % Ga doped ZnO thin film (Si^6+^ ion irradiated with 5 × 10^13^ ion fluence) for ethanol and acetone (250 ppm concentration) at 500 °C operating temperature.

Figure 21 shows the variation in gas response with variable ethanol and acetone gas concentration from 100 to 1000 ppm. It is evident from the figure that with increasing ethanol and acetone gas concentrations, the gas sensing response increases linearly. This is because the increasing gas concentration increases the surface reaction due to a larger surface area for the interaction of gas molecules^13,26^.

**Figure 22:**
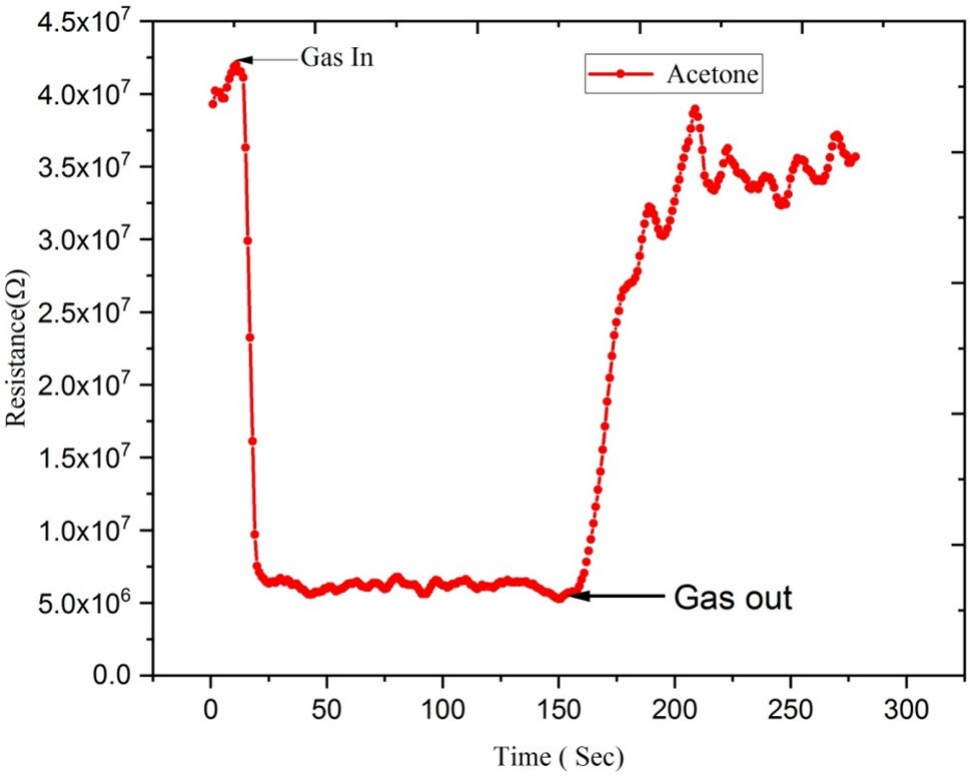
Variation of electrical resistance of Ga-doped ZnO thin film (Si^6+^ ion irradiated at 5 × 10^13^ ion fluence) with time for 250 ppm concentration of acetone gas.

**Figure 23:**
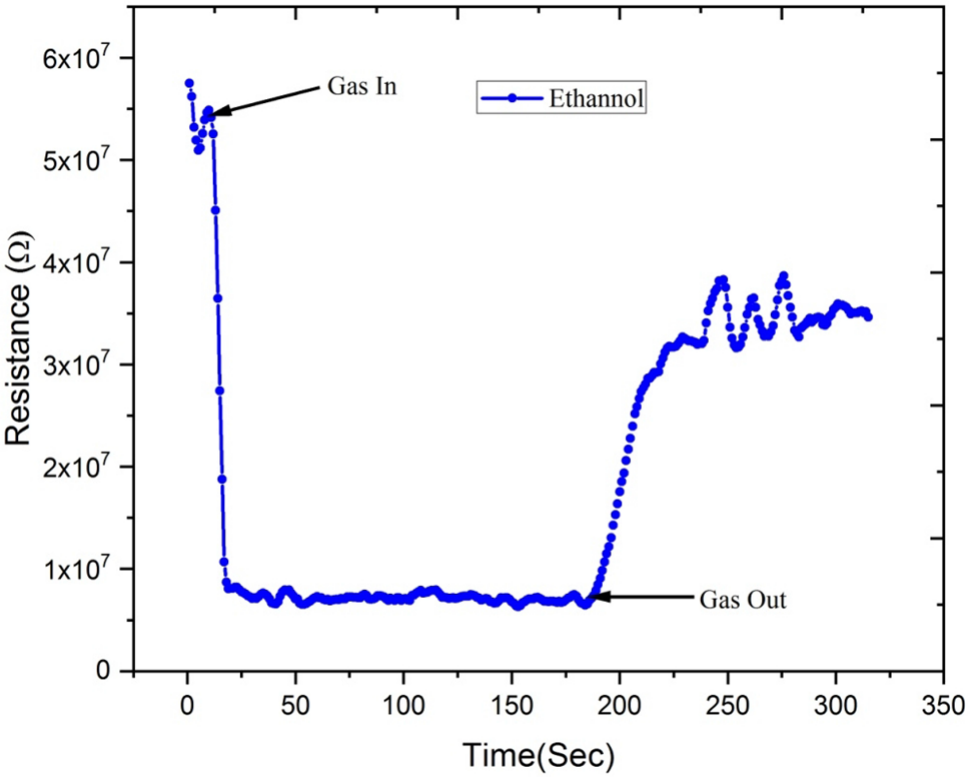
Variation of electrical resistance of Ga-doped ZnO thin film (Si^6+^ ion irradiated at 5 × 10^13^ ion fluence) with time for 250 ppm concentration of ethanol gas.

Figures 22 and 23 show the variation of electrical resistance of Ga-doped ZnO thin film irradiated with Si^6+^ ion irradiated at 5 × 10^13^ fluence with time for 250 ppm concentration of ethanol and acetone gas. In this case, with the exposure of ethanol gas, the resistance of Ga doped ZnO thin film has been decreased from 5.5 × 10^7^ to 1 × 10^7^ Ω, while with exposure of acetone gas the resistance has been decreased from 4.0 × 10^7^ to 5.0 × 10^6^ Ω. These results show that there is a large variation in the resistance of Ga doped thin film with exposure to acetone gas. It means the Ga doped ZnO sensor irradiated with Si^6+^ ion is more sensitive for acetone gas in comparison to ethanol gas.

**Figure 24:**
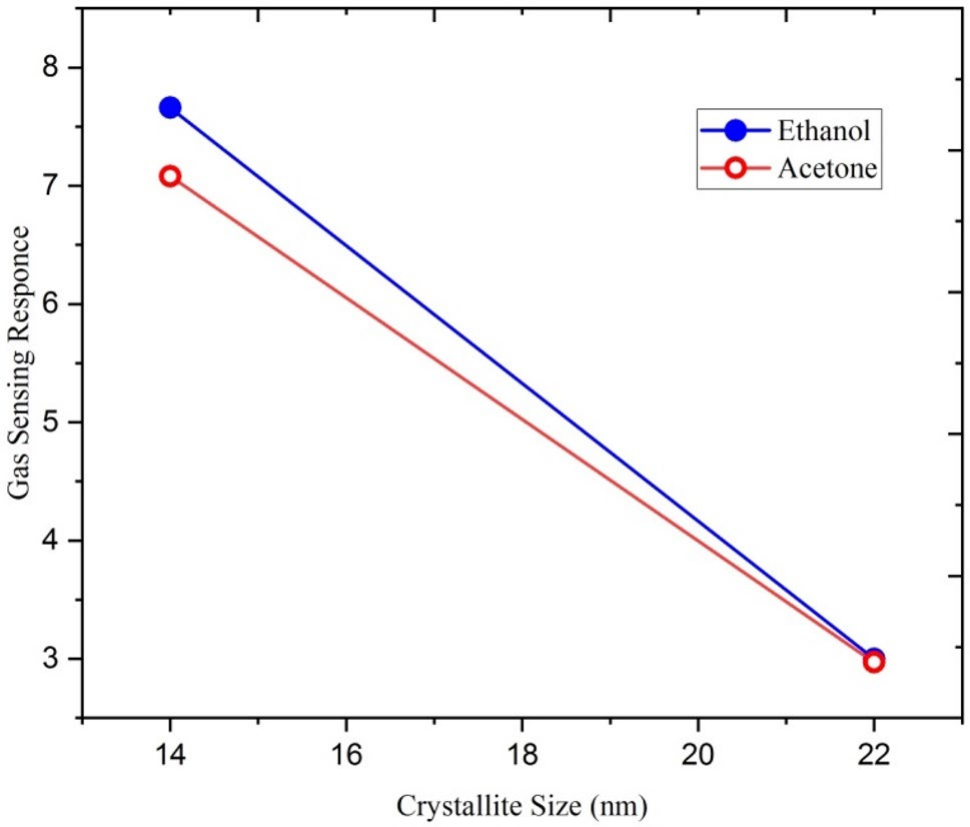
Variation of gas sensing response with the crystallite size.

Figure 24 shows the variation of ethanol and acetone gas sensing response of Ga doped ZnO thin film with crystallite size. It is clear from the figure that the sensing response of both ethanol and acetone gases increases with a decrease in crystallite size.

**Figure 25:**
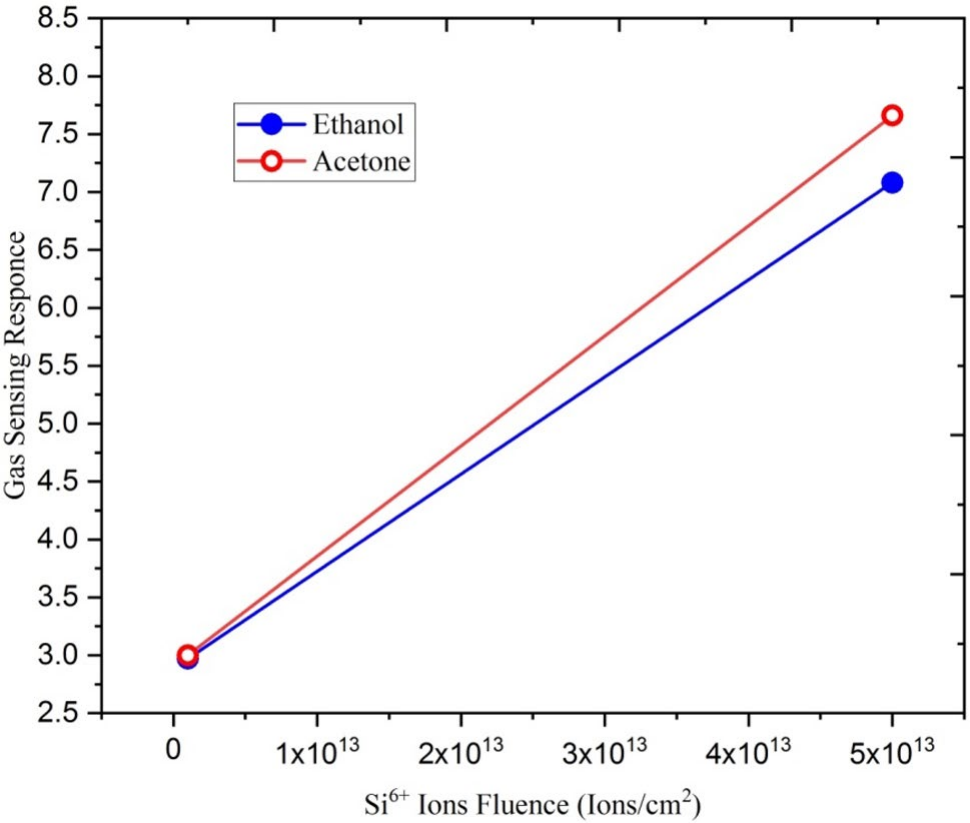
Variation of gas sensing response with Si^6+^ion fluence.

Figure 25 shows the variation of gas sensing response of Si^6+^ ion irradiated Ga doped ZnO thin film with silicon ion fluences. From figure, it is clear that with increasing ion fluence the gas sensing response increases.

The gas sensing response for Si^6+^ irradiated ZnO:Ga samples was found higher compared to the Ag^9+^ irradiated samples. The gas sensing response was found to increase with decrease in crystallite size. As observed from SEM, the decrease in crystallite size is more in Si^6+^ irradiated samples than in Ag^9+^ irradiated samples. This may be a reason for the high response of the films irradiated with Si^6+^ ions.

## Conclusions

In the present investigation, gas sensing characteristics of Ga doped ZnO thin film irradiated with silver and silicon ion beams were studied. The sensing response of ion beam irradiated Ga doped ZnO thin film shows that the ion beam irradiation has enhanced the gas sensing property of Ga doped ZnO thin film. The films irradiated with the silicon beam have higher response and short recovery times in comparison to the thin film irradiated with the silver ion beam. The sensing response of Ga doped ZnO thin film also increases with increasing the ion fluences. It is also concluded that the gas sensing response increases with a decrease in the crystallite size of the film.

## Data Availability

The datasets used and/or analysed during the current study available from the corresponding author on reasonable request.
